# Effect of Ultrasonic and Hydrothermal Treatment on Digestibility and Antioxidant Properties of Whole Wheat Flour with Different Amylose Content^§^

**DOI:** 10.17113/ftb.61.03.23.8016

**Published:** 2023-09

**Authors:** Valentina Nikolić, Slađana Žilić, Marijana Simić, Vesna Kandić, Primož Titan

**Affiliations:** 1 Maize Research Institute Zemun Polje, Slobodana Bajića 1, 11185 Belgrade-Zemun, Serbia; 2 Research Genetics and Agrochemistry Ltd., Krog, Brodarska 27, 9000 Murska Sobota, Slovenia

**Keywords:** whole wheat flour, ultrasonic treatment, hydrothermal treatment, *in vitro* digestibility, phenolic compounds, antioxidant capacity

## Abstract

**Research background:**

The consumption of whole grain cereal flour contributes to increased intake of dietary fibre and phenolic compounds beneficial to human health. However, whole grain flour also has some disadvantages, such as poor baking properties and lower technological quality. Applying ultrasonic and hydrothermal treatments can provide new opportunities to modify and improve the baking and biofunctionality of flour as well as the quality of baked goods.

**Experimental approach:**

The whole grain flour samples of six wheat varieties with different amylose content were studied. The original chemical composition and viscosity profiles of the flour were determined. The flour samples were subjected to ultrasonic treatment at a frequency of 30 kHz and temperature of 40 °C for 10 min and hydrothermal treatment on a magnetic stirrer with heating for 3 min after reaching the boiling point. The treatments were carried out to determine their influence on the studied digestible and antioxidant properties of the flour. A multistep *in vitro* enzymatic digestibility protocol simulating the digestion process in the human gastrointestinal tract was applied to the untreated and treated whole grain flour samples. Total free phenolic compound content and total antioxidant capacity were also determined.

**Results and conclusions:**

Hydrothermal treatment had a positive effect on the digestibility of the whole grain flour, especially in waxy wheat genotypes compared to those with high amylose content, due to the formation of resistant starch. The hydrothermal treatment had an overall negative effect on the antioxidant capacity of the flour samples, while ultrasonic treatment generally increased the analytical values of total free phenolic compounds by enhancing their extractability. These findings can provide valuable information for the development of new whole wheat foods.

**Novelty and scientific contribution:**

To the best of our knowledge, this type of study of the effects of ultrasound and hydrothermal treatment on the digestibility and antioxidant properties has not yet been performed on whole wheat flour with different amylose content. Waxy and high-amylose wheat varieties are considered novel raw materials because of their unique properties in bread making, such as improved bread texture and increased dietary ﬁbre content.

## INTRODUCTION

Wheat (*Triticum aestivum* L.) is one of the most important cereals consumed as a staple in a broad range of foods worldwide (*1*, *2*). The majority of wheat-based foods are made from reﬁned ﬂour, which lacks some essential nutrients that are lost during milling after bran and germ are removed (*3*). As epidemiological studies show (*4*), excessive consumption of refined grain foods is associated with an increase in health problems and diseases such as type 2 diabetes, coronary atherosclerosis, chronic cardiovascular disease, colon cancer and obesity. Whole wheat is rich in nutrients, particularly dietary fibre, B vitamins, minerals and phytochemicals such as phenolic acids, ﬂavonoids, carotenoids and tocopherols, all potentially beneficial to health (*1*). Most of these compounds are found in the aleurone layer, which gives this fraction the highest antioxidant activity, followed by bran and germ. The majority of the bioactive components of bran are bound to fibre, so they survive digestion in the gastrointestinal tract and enter the colon intact, where they create an antioxidant environment. However, studies have shown that the solubility and activity of bound phenolic compounds increases during digestion (*5*).

The field of grain processing is currently conducting more research on the potential applications of various thermal and non-thermal processes, including ultrasonic and hydrothermal treatments, to increase the bio- and baking functionality of flour and improve the final quality of baked goods (*6*). Ultrasound, for example, is a promising green approach that has found its application in food processing because it increases yield and enhances extraction rates of various bioactive compounds and nutrients, reducing water and energy waste. Ultrasound can either improve or degrade food quality depending on the processing variables (frequency, amplitude, treatment time) and food sources (*7*). The particular functional properties of wheat flour for food production, such as bread loaf volume, starch pasting and rate of starch digestion, are greatly influenced by the interactions between the two major macromolecular components, starch and protein (*8*). Starch granules are composed of two main components: amylose, a mainly linear polymer of (1→4)-linked α-d-glucopyranosyl units with some minor branching and amylopectin, a high-molecular-mass branched polymer with α-(1→4) and α-(1→6) linkages that form branch points (*2*, *9*). Because only α-glucosidic bonds are present in starch molecules, amylolytic enzymes produced by the human digestive system can break them down. However, the gelatinized cooked starch can convert to resistant starch after cooling, a form that is not hydrolysed by α-amylase and is not digested in the small intestine (*9*).

The physicochemical and functional properties of starch, the processing properties of flour, the digestibility and the edible quality of the final products are influenced by different amylose-amylopectin ratios and the structure of the starch granules (*2*, *10*). The proportions of amylose and amylopectin in starches vary depending on the cultivar. Normal wheat starches usually contain 22–35 % amylose and 65–78 % amylopectin, while waxy (amylose-free) wheat starches usually contain about 100 % amylopectin (*11*). Genotypes with an amylose content of 16–22 % can be classified as partially waxy according to Van Hung *et al.* (*12*). Waxy wheat is considered a novel raw material for the bakery sector due to its special quality in bread making, such as its ability to delay staling because starch is degraded less rapidly (*13*). Studies by Hung *et al.* (*13*) and Regina *et al.* (*14*) suggest that starches of ’high-amylose’ wheat genotypes, developed by reducing the activity of starch branching enzymes (SBE), have certain nutritional and health benefits due to slower digestion caused by the formation of resistant starch. Processing flour with high amylose content can produce more resistant starch than normal and waxy starch, allowing the development of novel food products with higher dietary fibre content (*15*, *16*).

In general, both internal (protein, amylose, dietary fibre and lipid content) and external factors (germination, processing, cooking and starch retrogradation) can affect the digestibility of a cereal-based food (*17*). The results of *in vitro* studies have shown that the amylose content, length distribution of amylopectin and amylose chains and starch granule shape are related to starch digestibility (*9*). The digestibility and absorption of nutrients such as starch in grains may also be delayed or even prevented in the small intestine by other components. These include protein and cell wall matrices that can be entrapped in starch granules and lipids that can form complexes with amylose, resulting in lower amounts of rapidly absorbed glucose (*18*).

**Fig. S1:**
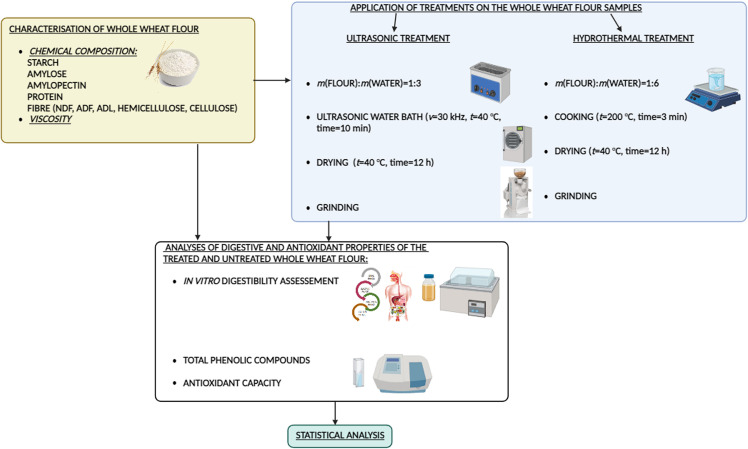
Schematic illustration of the processes applied in this study. NDF and ADF=neutral and acid detergent fibre, ADL=acid detergent lignin

The main focus of this study is to investigate the effect of ultrasonic and hydrothermal treatments on *in vitro* enzymatic digestibility, antioxidant capacity and total soluble free phenolic compound content of whole wheat flour with different amylose content. Prior to the treatments and simulation of the digestion processes, the chemical composition, total free phenolic compounds, antioxidant capacity and viscosity of whole wheat samples were evaluated for comparison and better statistical evaluation (Fig. S1).

## MATERIALS AND METHODS

### Chemicals

All chemicals and enzymes used in this study were of analytical and high performance liquid chromatography (HPLC) grade. The chemicals used were: ethanol 95–96 %, p.a. (Zorka Pharma-Hemija d.o.o, Šabac, Serbia), methanol 99.8 %, HPLC grade (JT Baker, Phillipsburg, NJ, USA), Folin-Ciocalteu phenol reagent (F9252) of analytical grade (0.67 M), ABTS (2,2′-azino-bis(3-ethylbenzothiazoline-6-sulfonic acid) diammonium salt (A1888-5G) (Sigma-Aldrich, Merck, St. Louis, MO, USA) and sodium carbonate, anhydrous, 99.5 %, p.a. (Loba Chemie, Pvt. Ltd, Mumbai, India). The enzymes used for the *in vitro* digestion protocol were all purchased from Sigma-Aldrich, Merck, namely: pepsin from porcine gastric mucosa (P7000-25G), bile extract porcine (B8631-100G), pancreatin from porcine pancreas (P1750-25G), protease from *Streptomyces griseus* (P5147-1G) and Viscozyme L cellulolytic enzyme mixture (V2010-50ML). Distilled water obtained in a laboratory distillation unit type D-8-SLO (Elektron, Banja Koviljača, Serbia) was used for the analyses.

### Plant material

Six genotypes of wheat (*Triticum aestivum* L.): Zemunska rosa, Apche, Titan SBE I, Titan SBE II, W1 and Waxy1, with different amylose content (0–36.5 %), genetic background and geographical origin were used in this study. All investigated genotypes were grown in the experimental field of the Maize Research Institute Zemun Polje, Belgrade, Serbia (44˚52´N, 20˚19´E, 81 m ASL), during the 2021/2022 growing season. Standard cultivation practices were followed to provide adequate nutrients to the plants and to keep the plots free from diseases and weeds. Whole wheat flour was obtained by milling in a laboratory mill (Perten MILL 120 CE; Perten Instruments, Hägersten, Sweden) for fine sample preparation (mesh size 0.5 mm).

### Ultrasonic treatment

Whole wheat flour samples (10 g) were mixed with water in a mass ratio (hydromodulus) of 1:3, placed in glass flasks and subjected to ultrasonic treatment, which was performed in an ultrasonic water bath (model: UZ 4P 220/115V; power 100W; Iskra, Ljubljana, Slovenia) at a frequency of 30 kHz and a temperature of 40 °C for 10 min. After ultrasonic treatment, the mixtures were transferred to Petri dishes and dried overnight in a forced-air oven (Memmert UF 55; Memmert GmbH + Co. KG, Schwabach, Germany) at 40 °C and then ground in a laboratory mill (Perten MILL 120 CE; Perten Instruments). The dry matter content of the samples was determined by the conventional drying method at 105 °C to a constant mass. The experiments were conducted in duplicate.

### Hydrothermal treatment

Whole wheat flour samples (10 g) were mixed with water in a mass ratio (hydromodulus) of 1:6 in glass flasks. The magnet was added to the mixture and placed on a preheated hot plate (*t*=200 °C) with magnetic stirrer (ARE heating magnetic stirrer; VELP Scientifica, Usmate Velate MB, Italy) and subjected to hydrothermal treatment. The samples were boiled for 3 min after reaching the boiling point, while being continuously stirred manually with a glass stirring rod. The mixtures were transferred to Petri dishes and dried overnight in a forced-air oven (Memmert UF 55; Memmert GmbH + Co. KG) at 40 °C and then ground in a laboratory mill (Perten MILL 120 CE; Perten Instruments). The dry matter content of the samples was determined by the conventional drying method at 105 °C to a constant mass. The experiments were conducted in duplicate.

### Analysis of protein and starch in the untreated whole wheat flour

The Kjeldahl method was used to determine the protein content on the BÜCHI Kjeldahl System (AutoKjeldahl distillation unit K-350 and speed digester K-439; BÜCHI Labortechnik, Flawil, Switzerland), with total nitrogen multiplied by factor 5.7 used for wheat and wheat flour (*19*). The Ewers method (*20*) was used to determine the starch content with a polarimeter (UniPol L 2020; Schmidt + Haensch GmbH & Co., Berlin, Germany). According to the colorimetric method developed by McGrance *et al.* (*21*), the amounts of amylose and amylopectin were calculated after measuring the absorbance of the samples with a spectrophotometer (Agilent 8453 UV-visible spectroscopy system; Agilent Technologies, Inc, Santa Clara, CA, USA) at 600 nm. All results are expressed as mass fraction in % on dry mass basis. The analyses were performed in two replicates.

### Analysis of dietary ﬁbre in the untreated whole wheat flour

Using the hot extraction device Fibertec system FOSS 2010 (FOSS Tecator, Hoeganaes, Sweden), the Van Soest detergent method modified by Mertens (*22*) was applied to quantify the mass fractions of hemicellulose, cellulose and lignin (ADL). Cellulose mass fraction was calculated as the difference between mass fractions of acid detergent fibre (ADF) and lignin, while hemicellulose mass fraction was determined as the difference between mass fractions of neutral detergent fibre (NDF) and ADF. Each result is expressed as *w*/% on dry mass basis. The analyses were performed in two replicates.

### Pasting properties of the untreated whole wheat flour

The changes in apparent viscosity of aqueous suspensions were analysed to obtain the pasting curves of the whole wheat flour studied. Suspensions containing 8 % starch (total mass 500 g) were heated in a viscograph (model PT 100; C.W. Brabender Instruments, Inc, Duisburg, Germany) from 25 to 95 °C at a rate of 1.5 °C/min. The suspensions were thermostatted at 95 °C for 30 min, then cooled to 50 °C and held for a further 10 min. The analyses were performed in two replicates according to the official method (*23*) and the viscosity was expressed in Pa·s.

### Extraction of total soluble free phenolic compounds from untreated and thermally treated whole wheat flour

Extracts for the detection of total soluble free phenolic compounds were obtained by continuous shaking of 0.5 g of whole wheat flour sample in 10 mL of *φ*(ethanol)=70 % for 30 min at room temperature on a horizontal shaker type Thys 2 (VEB MLW Labortechnik, Ilmenau, Germany). After centrifugation at 11 200×*g* for 5 min (Velocity 18R refrigerated benchtop centrifuge; Dynamica, Livingston, UK), the supernatant was used for the detection of total phenolic compounds. A volume of 5 mL of the extracts was evaporated to dryness under the N_2_ stream at 30 °C (Reacti-Therm nitrogen evaporator system 18821; Thermo Fisher Scientific Inc., Waltham, MA, USA) and the final residues were dissolved in methanol (1.5 mL). Prior to analyses, the extracts were stored at -70 °C. All extractions were performed in duplicate.

### Analysis of total soluble free phenolic compounds in untreated and thermally treated whole wheat flour

Total soluble free phenolic content was determined by the Folin–Ciocalteu assay and expressed as mg of gallic acid equivalent (GAE) per kg of dry mass (*24*). A volume of 50 to 150 µL of the extract was transferred to the test tubes and made up to 500 µL with water. After adding Folin–Ciocalteu reagent (250 µL of 0.2 M) and 1.25 mL of 20 % aqueous Na_2_CO_3_ solution to the tubes, the samples were vortexed (VORTEX ZX3; VELP Scientifica Srl, Usmate, Italy). The absorbances of the mixtures were measured (Agilent 8453 spectrophotometer; Agilent Technologies, Inc.) after 40 min at 750 nm. The analyses were performed in duplicate.

### Analysis of total antioxidant capacity of untreated and thermally treated whole wheat flour

The antioxidant capacity of whole wheat flour was measured by the direct or QUENCHER method using the ABTS (*25*). Absorbance was measured at 734 nm (Agilent 8453 spectrophotometer; Agilent Technologies, Inc.) and total antioxidant capacity was expressed in mmol Trolox equivalents (TE) per kg of dry mass. The analyses were done in two replicates.

### In vitro multistep enzymatic digestion protocol

To determine the potential digestibility of whole wheat flour for human consumption as a function of processing conditions, a multistep *in vitro* digestion method was used. The method, consisting of an oral, gastric, duodenal and colon phase (*26*, *27*), was performed without attempting to fully mimic gastrointestinal digestion. Samples obtained after *in vitro* digestion were filtered through qualitative filter paper, air-dried in a ventilated oven for 2 h and then dried to constant mass at 105 °C for 4 h. The samples were weighed and digestibility was calculated according to the following equation:

Digestibility=((*m*_0_-*m*_d_)/*m*_0_)·100 /1/

where *m*_0_ is the mass of absolutely dry sample before digestion and *m*_d_ is the remaining (undigested) mass of absolutely dry sample.

### Statistical analysis

Data were expressed as a mean±standard deviation of two independent replicates (*N*=2). Statistical analyses were performed using Minitab statistical software, v. 19.2.0 (*28*). The signiﬁcance of differences between samples was analysed using the Tukey’s test. Differences between the means with probability p<0.05 were accepted as statistically significant. Principal component analysis (PCA) was used to visually represent the relationships between the observed parameters. A positive correlation between two parameters was represented by an acute angle between them, while a negative correlation was represented by an obtuse angle.

## RESULTS AND DISCUSSION

### Chemical composition and viscosity profiles of untreated whole wheat flour

The initial chemical composition and fibre content of the studied whole wheat flour are listed in Table 1.

**Table 1:** Chemical composition of untreated whole wheat flour samples (before thermal treatment)

	Genotype					
	Zemunska rosa	Apache	Titan SBE I *w*/%	Titan SBE II	W1	Waxy1
Starch	(67.74±0.5)^b^	(69.34±0.5)^a^	(64.10±0.00)^d^	(58.78±0.3)^e^	(69.15±0.00)^a^	(66.07±0.00)^c^
Amylose	(19.00±0.00)^c^	(13.90±0.5)^d^	(36.50±0.5)^a^	(28.00±0.5)^b^	(0.00±0.00)^e^	(0.00±0.00)^e^
Amylopectin	(81.00±0.00)^c^	(86.01±0.5)^b^	(63.50±0.5)^e^	(72.00±0.5)^d^	(100.00±0.00)^a^	(100.00±0.00)^a^
Protein	(11.32±0.04)^e^	(12.10±0.2)^d^	(14.87±0.06)^b^	(16.23±0.1)^a^	(12.58±0.1)^d^	(14.21±0.2)^c^
NDF	(73.26±0.3)^a^	(67.06±0.3)^b^	(63.66±0.1)^c^	(68.54±0.04)^b^	(48.13±0. 6)^d^	(66.86±0.7)^b^
ADF	(6.32±0.6)^b^	(4.53±0.07)^c^	(10.39±0.3)^a^	(10.08±0.4)^a^	(5.24±0.3)^bc^	(10.70±0.5)^a^
ADL	(3.49±0.08)^b^	(2.65±0.4)^bc^	(7.37±0.6)^a^	(1.87±0.0)^c^	(2.14±0.2)^c^	(6.64±0.1)^a^
Hemicellulose	(66.94±1.0)^a^	(62.53±0.4)^b^	(53.27±0.4)^e^	(58.47±0.4)^c^	(42.89±0.2)^f^	(56.16±0.2)^d^
Cellulose	(2.84±0.7)^b^	(1.89±0.4)^b^	(3.02±0.9)^b^	(8.21±0.4)^a^	(3.11±0.1)^b^	(4.06±0.5)^b^
Values are presented as mean±standard deviation (*N*=2). Mean values followed by the same letter within the same row are not significantly different (α=0.05 %). SBE=starch branching enzyme, NDF=neutral detergent fibre, ADF=acid detergent fibre, ADL=acid detergent lignin						

**Fig. 1:**
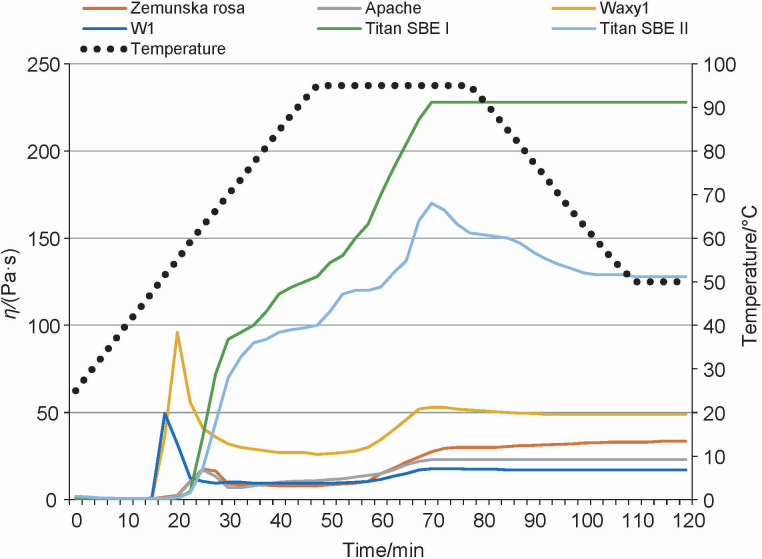
Pasting profiles (viscosity curves) of untreated whole wheat flour samples (before the treatments). SBE=starch branching enzyme

The total starch mass fraction ranged from 64.10 % (Titan SBE I) to 69.34 % (Apache). Based on the amylose mass fraction, the wheat genotype Apache (13.90 %) could be classified as normal, while Zemunska rosa with a typical amylose mass fraction of 19.00 % could be subcategorised as “partially waxy”. Previous studies have shown that partially waxy wheat (16-22 % amylose) is best suited for the production of noodles traditionally used in Asian cuisine (*12*). The two genotypes examined in this study, Titan SBE I and Titan SBE II, obtained by reducing the activity of starch branching enzymes (SBE I and SBE II), had 36.50 and 28.00 % amylose, respectively. The highest amylose-to-amylopectin ratio, an indicator of a low glycaemic index, was found in the Titan SBE I genotype (0.57), which is rich in amylose. The results show that the Titan SBE I genotype can be classified as “rich in amylose”, as it contained more than 35 % amylose, while the Titan SBE II genotype did not have an increased amylose mass fraction. Nevertheless, the pasting curve of Titan SBE II in Fig. 1 shows the transitional properties of this genotype, which lie in the range between normal and amylose-rich starch. The starch of the wheat genotypes Waxy1 and W1 did not contain amylose, *i.e*. it consisted of 100 % amylopectin. The mass fraction of total protein, the second most abundant macronutrient in wheat, determined in the whole wheat flour samples varied considerably among the genotypes, ranging from 11.32 % (Zemunska rosa) to 16.23 % (Titan SBE II) (Table 1). These results indicate that wheat with high amylose content has on average the highest protein mass fraction, followed by waxy wheat and wheat with average amylose content and are consistent with previously reported results (*29*, *30*). Proteins, including non-gluten (15-20 %) and gluten proteins (80-85 % of total wheat protein), play a key role in determining the suitability of wheat flour for processing into various food products by affecting their functional properties (*8*). Similar total protein contents determined on bread and durum wheat varieties have been reported previously (*8*, *31*). The results of Li *et al.* (*32*) suggest that the increased protein content in native high amylose wheat flour, which our results also confirm, and the thermal stability of starch in noodles prepared with this type of flour result in lower digestibility and consequently increase the resistance to α-amylase digestion. Waxy wheat varieties are considered responsible for the longer shelf life of baked products, without using wheat gluten. However, high amylose wheat varieties have been produced with significantly higher amylose content (55–65 %) depending on the enzyme that controls amylose synthesis (*33*).

In addition, significant differences in the fibre composition of the studied genotypes were found (Table 1). The flour of the genotype with an average amylose mass fraction, Zemunska rosa with the highest insoluble fibre mass fraction, *i.e. w*(NDF)=73.26 %, also had a very high mass fraction of hemicellulose (β-glucans and arabinoxylans) (66.94 %). On the other hand, the waxy wheat genotype W1 had the lowest mass fractions of all the dietary fibres analysed, which was not the case for the Waxy1 genotype, which had a significantly higher fibre mass fraction. Morita *et al.* (*29*) reported higher fibre content in flour from high amylose and waxy wheat genotypes than in average wheat flour. According to our results, high amylose Titan SBE I and Waxy1 genotypes had the highest insoluble lignin (ADL) fibre mass fraction.

The apparent viscosity of the aqueous flour suspensions varied, as shown by the pasting curves in Fig. 1.

The genotypes with lower amylose mass fraction such as Apache (13.90 %) and Zemunska rosa (19.00 %) showed a similar trend in the development of peak and final viscosity. The high activity of α-amylase, which causes the enzymatic breakdown of starch molecules in the endosperm to simple sugars, may have resulted in low viscosity. This type of flour, usually produced by milling sprouted wheat, has a lower gas retention capacity and could affect the baking ability of bread (*34*). The transition between flour with an average and a high amylose mass fraction can be seen in the pasting curve of Titan SBE II (28.00 % amylose). The pasting curve of the amylose-rich Titan SBE I genotype (36.50 % amylose) showed a constant increase when heating or thermostating the aqueous flour suspensions without reaching the peak viscosity. The peak viscosity was reached relatively quickly; breakdown and final viscosity were very high. The flour of the genotypes Waxy1 and W1 showed a pasting curve typical for waxy wheat. According to Blazek and Copeland (*35*), the pasting curves of waxy wheat genotypes are often characterised by high peak viscosities and low final viscosities compared to wheat flour with an average amylose content, which is in agreement with our results. The results are in line with previous findings (*31*).

### Effect of thermal treatments on in vitro digestibility of the whole wheat flour

**Fig. 2:**
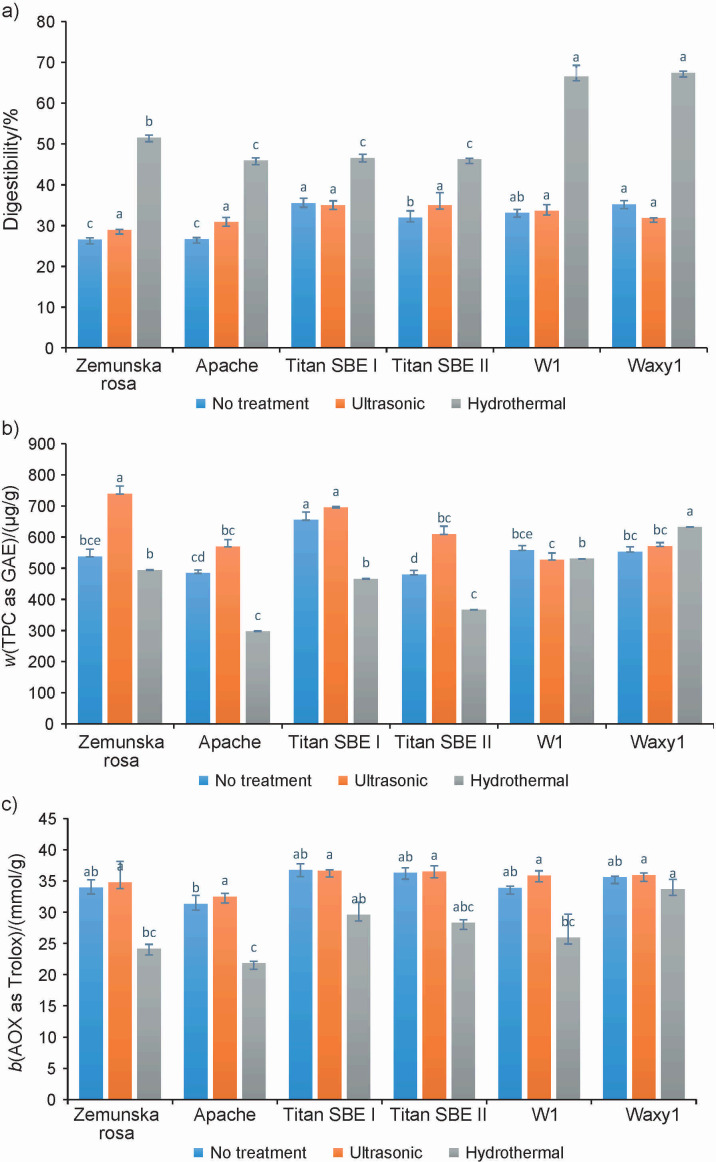
The effect of thermal treatment on: a) digestibility, b) total soluble free phenolic compounds (TPC) expressed as gallic acid equivalents (GAE) and c) antioxidant capacity (AOX) of whole wheat flour expressed as Trolox equivalents on dry mass basis. Values are presented as mean±standard deviation (*N*=2). Means followed by the same letter within the same genotype are not significantly different (α=0.05 %). SBE=starch branching enzyme

The results of the multistep enzymatic *in vitro* digestion protocol are shown in Fig. 2a.

The digestibility of the untreated samples ranged from 26.55 % in Zemunska rosa, a genotype with a typical amylose mass fraction, to 35.49 % in Titan SBE I, a genotype with high amylose mass fraction. After the ultrasonic treatment, the highest increase in digestibility compared to the untreated same flour sample was observed in the whole wheat flour of the Apache genotype (about 13.5 %), while Waxy1 amylopectin genotype showed a significant decrease in digestibility (about 9 %). Furthermore, there was no linear correlation between ultrasonic treatment and the *in vitro* digestibility of the samples (Fig. 2a). Moreover, the digestibility of whole wheat flour of genotypes W1 and Titan SBE I remained unchanged after ultrasonic treatment compared to untreated flour (33.56 vs 33.08 % and 34.98 vs 35.49 %, respectively).

In contrast, the hydrothermally treated samples of whole wheat flour all showed a high increase in digestibility compared to the untreated same flour sample. Flour from waxy genotypes showed the highest increase in digestibility (W1 showed a double increase and Waxy1 showed a 1.9-fold increase), followed by standard wheat varieties (Zemunska rosa 1.9-fold and Apache 1.7-fold), while wheat genotypes with high amylose content showed the lowest increase in digestibility (Titans SBE II 1.4-fold and Titan SBE I 1.3-fold). The limited increase in digestibility of flour obtained from SBE genotypes can be explained by the formation of resistant starch, which occurs during cooling with retrogradation of starch (*17*). However, the content of resistant starch was not measured in our study. The effect of hydrothermal treatment on the improved digestibility of whole wheat flour can be attributed in large part to gelatinisation of the starch present in large quantities in this material, making it more readily available for enzymatic degradation (*36*). Similarly, a study by Kiers *et al.* (*37*) showed that after cooking and subsequent malt saccharification, the mass fraction of digestible white maize increased about threefold, from 25.5 to 63.6 %, while cooking increased the total digestibility of soybeans from 36.5 to 44.8 % and the digestibility of cowpea from 15.4 to 40.9 %. Considering that the hydration pattern of any substrate plays a crucial role in enzymatic processes and hence digestibility, it is possible that soft wheat varieties with a better hydration pattern are more digestible than the hard varieties. Even though endosperm texture depends on the genetic background, the interaction between genotype and environment is of great importance. Endosperm texture cannot be categorically described as ’hard’ or ’soft’, but lies somewhere along this spectrum. The protein mass fraction of durum wheat is around 15 %, while that of soft wheat varieties is closer to 10 % (*38*). Although durum wheat, as a representative of durum wheat varieties, was not included in our trial, the results confirm that the flour of the high-protein and high-amylose genotypes Titan SBE I and Titan SBE II generally has lower digestibility after cooking (46.58 and 46.21 %, respectively) than the flour of the Zemunska rosa and waxy wheat varieties (51.56, 66.54 and 67.43 %, respectively) with lower protein mass fraction. Besides the protein mass fraction, the content and structure of starch in the grain has also play a decisive role in the digestibility of the grain/wholemeal flour. Due to their stable and ordered semi-crystalline structure, starch granules are not water-soluble at room temperature. However, when native starch is subjected to thermal treatments such as cooking, α-amylase inhibitors and α-amylase itself are denatured, the ordered structure is disrupted and various processes such as swelling of the granules, leaching of the amylose and disorganisation of amylopectin take place (*38*). Research has shown that hydrothermal treatments such as heat-moisture treatment and annealing can increase the slowly digestible and resistant starch fraction in starch from various plant sources without destroying the granular structure. Although a reduction in starch digestibility would presumably have to be accompanied by a reduction in the protein digestibility because the starch/protein matrix is destroyed, there does not seem to be any evidence for this (*38*). Foods with a higher amylose content retrograde faster and to a greater extent, even if the crystalline structure is broken when heated. Our results showed that the flour with 100 % amylopectin starch was on average 30 % more digestible after ultrasonic treatment than the flour with 36 and 28 % amylose. When the amylose content is increased, more dietary resistant starch is formed (*33*). In addition, lipids may aggregate with more amylose or longer branching chains when heated and cooled. Wu *et al.* (*39*) showed that phenolic compounds attach to macromolecules in food such as proteins, lipids and carbohydrates and block the digestive enzymes (α-amylase and amyloglucosidase) through chemical interactions, causing the enzymes to precipitate and reducing their activity in digesting carbohydrates. Aggregates of polymeric glycoside complexes form between phenolic compounds and sugars, which can interfere with the absorption of phenols. By delaying carbohydrate digestion and prolonging digestion time, the interaction between polyphenols and carbohydrates can reduce the release and absorption of glucose (*40*).

### Effect of thermal treatments on the content of total soluble free phenolic compounds in whole wheat flour

Phenolic compounds are found predominantly in bran, which is thus responsible for the total antioxidant capacity of wheat (*5*). The majority of phenolic compounds (about 95 %) are bound to the cell wall polysaccharides and are called dietary fibre phenolic compounds (*41*). In our study, the content of free, soluble phenolic compounds was measured. The changes in phenolic compounds before and after thermal treatment are shown in Fig. 2b. Among the untreated whole wheat samples, the Titan SBE I genotype with high amylose mass fraction had the highest total free phenolic mass fraction (expressed on dry mass basis in GAE, 655.26 μg/g), while the Titan SBE II genotype had the lowest mass fraction of these bioactive components (479.99 μg/g). A study by Adom *et al.* (*42*) reported that the free phenolic content in the untreated flour of wheat varieties ranged from 119.61 to 201.25 μmol/100 g of grain and that the ratio of bound to free phenolic content was on average 2.5 to 5.4 times higher in the wheat genotypes studied. The ultrasonic treatment had a positive effect on the total soluble free phenolic compound mass fraction in all whole wheat samples, except for the amylopectin genotype W1. Conversely, the soluble free phenolic mass fraction decreased during cooking in all samples except Waxy1. Compared to the untreated wheat flour, the mass fraction of free phenolic compounds, which can be described as bioavailable, was increased by about 27, 15, 6, 21 and 3 % after ultrasonic treatment of Zemunska rosa, Apache, Titan SBE I, Titan SBE II and Waxy1 flours, respectively. This result can be attributed to the hydrolysis of the bound phenolic compounds, which contributes to the increase in free phenolic content after hydrothermal treatment. On the other hand, the loss of soluble free phenolic compounds due to thermal degradation during cooking at about 80 °C for 3 min was 8, 39, 21, 23 and 5 % for Zemunska rosa, Apache, Titan SBE I, Titan SBE II and W1 flours, respectively. Some studies reported that bioactive compounds, including anthocyanins and ascorbic acid, were degraded after prolonged ultrasonic treatment, while heat treatment and extrusion showed different effects in barley, rye, triticale, oats, sorghum and millet (*43*). Cui *et al.* (*43*) reported that despite an increase in the first two hours, the phenolic compounds started to degrade in a time-dependent manner. Sęczyk *et al.* (*44*) reported that the hydrothermal treatment had a negative effect on the phenolic content of the flour samples studied, compared to their initial value. Our results also show that the flour of the waxy genotypes was the most thermally stable in terms of soluble free phenolic compound content.

### Effect of thermal treatments on the antioxidant capacity of the whole wheat flour

The changes in antioxidant capacity determined before and after the ultrasonic and hydrothermal treatments are shown in Fig. 2c. The results show that antioxidant capacities did not vary significantly among wheat genotypes. However, the average antioxidant capacity of the untreated flour of bread wheat genotypes (Zemunska rosa and Apache) was the lowest and was, expressed in TE, 32.64 mmol/kg. The average antioxidant capacity of flour of high-amylose wheat and waxy wheat genotypes was 36.59 and 34.75 mmol/kg, respectively. Žilić *et al.* (*5*) reported values of antioxidant capacity of bran and debranned wheat flour obtained from different bread and durum wheat varieties ranging from 6.78 to 19.55 and from 27.81 to 37.57 mmol/kg, respectively, suggesting that most of the antioxidant potential of the grain is concentrated in the bran. In addition, Adom *et al.* (*42*) found that the bound phytochemicals accounted for an average of 82 % of the total antioxidant activity in 11 wheat varieties studied. According to our study, ultrasonic treatment increased the antioxidant capacity of the flour, although not statistically significantly. In a study by Cui *et al.* (*43*), the relationship between thermal treatment and antioxidant capacity was also inconclusive. The slight increase in total antioxidant capacity of wheat flour after ultrasonic treatment could be explained by the increased release of bound phenolic compounds mentioned above. The results of Žilić *et al.* (*45*) showed that the phenolic compounds of wheat grain in bound form, which dominate in the analysed extracts, have a lower antioxidant capacity than the hydrolysed and isolated free forms. In contrast to ultrasonic treatment, the antioxidant capacity of flour samples after cooking was reduced by about 28, 30, 20, 22, 23 and 5 % compared to untreated flour of Zemunska rosa, Apache, Titan SBE I, Titan SBE II, W1 and Waxy1 genotypes, respectively. In these samples, the antioxidant capacity correlates very well with a reduction in the content of total soluble free phenolic compounds in them.

### Principal component analysis

**Fig. 3:**
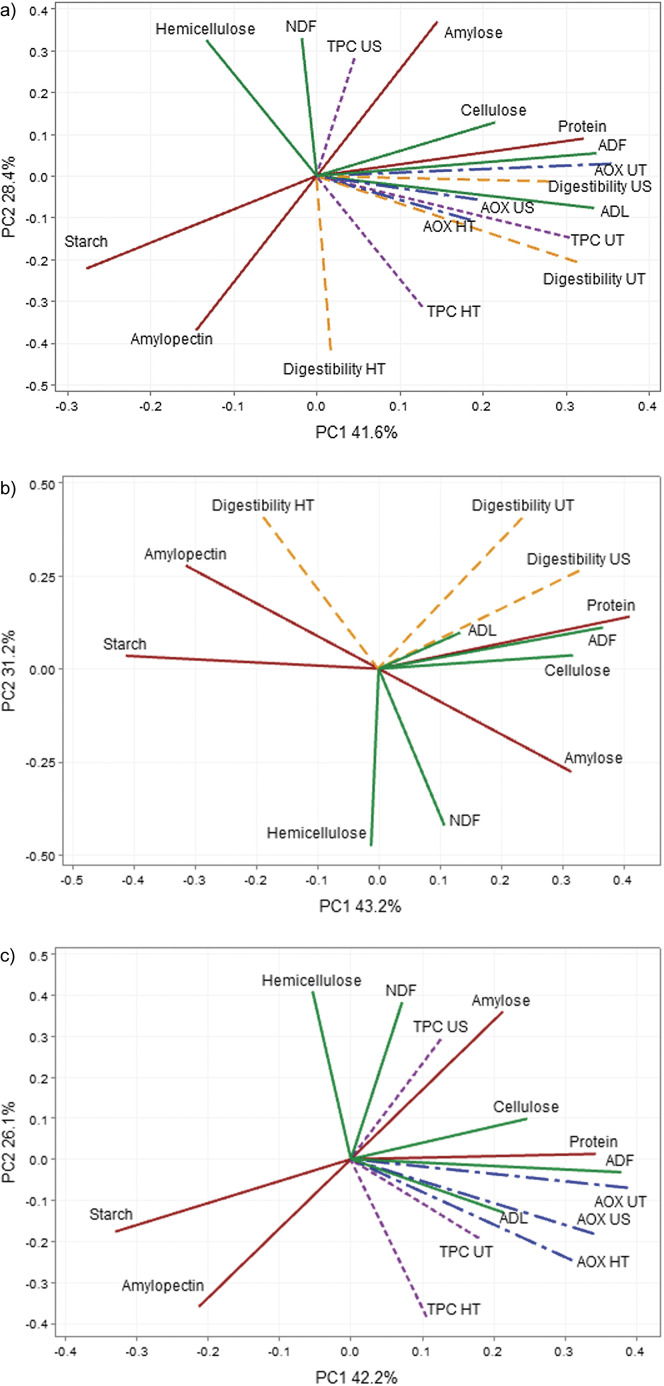
Genotype by trait bi-plot showing the interrelationship between: a) chemical composition, digestibility and antioxidant properties of untreated and thermally treated whole wheat flour, b) chemical composition and digestibility of untreated whole wheat flour after thermal treatment and c) chemical composition and antioxidant properties of untreated whole wheat flour after thermal treatment. NDF=neutral detergent fibre, ADF=acid detergent fibre, ADL=acid detergent lignin, TPC=total soluble free phenolic compounds, AOX=antioxidant capacity, UT=untreated, US=ultrasonic treatment, HT=hydrothermal treatment

The graphical representation of the results of principal component analysis is shown in Fig. 3.

Principal component analysis showed that amylose content was highly negatively correlated with digestibility after hydrothermal treatment. Conversely, the amylopectin content was strongly positively correlated with this parameter. Starch content negatively influenced digestibility after ultrasonic treatment. Protein content was very strongly correlated with digestibility after ultrasonic treatment, in a significant correlation with the digestibility of the untreated flour samples. In contrast, no significant correlation was found between the digestibility of hydrothermally treated (cooked) samples and the protein content. The ADF content showed a significant correlation with the antioxidant capacity especially in the untreated whole wheat flour samples, followed by the cooked and ultrasonically treated samples. A correlation between protein content and antioxidant capacity of the untreated and, to a lesser extent, the treated samples was also found.

## CONCLUSIONS

This study highlights some of the advantages and disadvantages of different treatments on the digestive and antioxidant properties of whole wheat flour. Hydrothermal treatment had a positive effect on the digestibility of the whole wheat flour types, especially in waxy genotypes compared to those with high amylose content, which can be explained by the formation of resistant starch. Hydrothermal treatment had an overall negative effect on the antioxidant capacity of the flour samples, while ultrasound generally had a positive effect on the total free phenolic compound content. The application of ultrasonic and hydrothermal treatments could provide new opportunities for the modification and improvement of the baking and biofunctionality of flour types as well as the quality of baked goods. Currently, further research is needed to fully understand how various pretreatments, components of a food's complex structure, interactions between macronutrient contents and digestibility affect the antioxidant properties of whole wheat flour. In this regard, the results obtained in this study may be useful for further studies focusing on the effects of pretreatments on the biofunctional properties of whole grain flour types.
